# Association between perioperative rate pressure product and postoperative delirium in geriatric patients with hip fracture

**DOI:** 10.3389/fmed.2025.1651278

**Published:** 2025-10-28

**Authors:** Ning Kang, Ying Deng, Ning Yang, Zhengqian Li, Zhongshen Kuang, Yi Yuan, Xiangyang Guo

**Affiliations:** ^1^Department of Anesthesiology, Peking University Third Hospital, Beijing, China; ^2^Department of Intensive Care Unit, Zhanjiang Central People's Hospital, Zhanjiang, Guangdong, China; ^3^Department of Anesthesiology, Beijing Jishuitan Hospital, Beijing, China; ^4^Beijing Center of Quality Control and Improvement on Clinical Anesthesia, Beijing, China

**Keywords:** geriatric patients, hip fracture, postoperative delirium, rate pressure product, inflammation, interleukin-6

## Abstract

**Introduction:**

Postoperative delirium (POD) is a serious complication of elderly hip fracture patients, leading to deleterious outcomes and substantial healthcare burdens. Early predictors remain a critical imperative. Rate pressure product (RPP), a stress indicator, has not been studied in relation to POD.

**Objective:**

This study aimed to investigate the relationship between RPP at admission and incidence of POD in elderly patients undergoing hip fracture surgery and to explore whether the effect of RPP on POD is mediated by inflammatory response.

**Methods:**

This study was conducted on patients aged ≥65 years who underwent hip fracture surgery under spinal anesthesia. POD was diagnosed using the 3-Minute Diagnostic Interview for CAM-defined Delirium (3D-CAM). A comprehensive dataset including demographics, clinical parameters, Mini-Mental State Examination (MMSE) scores, and RPP. Preoperative plasma levels of IL-1βand IL-6 were quantified. To mitigate confounding biases, a propensity score matching (PSM) was performed. Logistic regression analyses were used to build a model predicting probability of POD. Receiver operating characteristic (ROC) curve analysis assessed the predictive utility of RPP. Mediation analysis was employed to further explore the relationship between RPP and POD.

**Results:**

From an initial pool of 468 elderly patients undergone hip fractures, the rigorous screening and matching process culminated in a final analytical cohort of 150 patients. Post-PSM, patients in POD group exhibited higher admission RPP (*p* < 0.001), and elevated preoperative plasma IL-6 levels (*p* < 0.001) compared with patients in non-POD group. The binary logistic regression pinpointed higher admission RPP (OR: 1.325, 95% CI: 1.098–1.599, *p* = 0.003) and elevated preoperative plasma IL-6 (*p* < 0.001) as potent and independent risk factors of POD. Admission RPP demonstrated a commendable ability to predict POD, yielding an AUC of 0.727 (95% CI, 0.639–0.815, *p* < 0.001). Moreover, the results of mediation analysis show that the effect of RPP at admission on POD may be partially mediated by preoperative plasma IL-6.

**Conclusion:**

Elevated RPP at admission is a risk factor of POD in elderly patients undergoing hip fracture surgery and the effect of RPP at admission on POD may be partially mediated by preoperative plasma IL-6.

## 1 Introduction

Postoperative delirium (POD) is a devastating complication in elderly patients undergoing hip fracture surgery, with incidence ranging from 4 to 53.3% (1). Hip fractures number is projected to increase from 258,000 in 2010 to 289,000 in 2030 in America alone, and this rising incidence is expected to be mirrored by a concurrent increase in POD cases (2). This acute state of cerebral dysfunction, characterized by fluctuating disturbances in attention, awareness, and cognition, is far from a transient, benign inconvenience (3). Instead, POD is intrinsically linked to a grim litany of adverse outcomes: prolonged hospital stays that stretch into weeks, an increased likelihood of institutionalization, a disheartening decline in functional independence, significantly elevated risks of morbidity and mortality, and, quite distressingly, an accelerated trajectory toward long-term cognitive impairment and dementia (4). The profound human and economic costs associated with POD underscore the urgent, almost desperate, need for robust strategies aimed at early identification and mitigation (5). Despite decades of research, the precise pathophysiological mechanisms underpinning POD remain incompletely unraveled (3). The surgical trauma itself, coupled with anesthesia, pain, infection, and metabolic derangements, is thought to trigger a neuroinflammatory cascade and neurotransmitter imbalances, particularly in brains already rendered vulnerable by age-related changes or pre-existing cognitive frailty (6, 7). Therefore, exploring predictive biomarkers of POD is of great significance. Early recognition of at-risk individuals could, theoretically, pave the way for tailored interventions designed to attenuate the risk or severity of this debilitating condition (6).

Rate pressure product (RPP), the simple product of heart rate and systolic blood pressure, has garnered interest as an indirect, yet insightful, index of myocardial oxygen demand and, by extension, systemic sympathetic tone (8). An elevated RPP upon hospital admission might signify a state of heightened physiological stress or compromised cardiovascular reserve even before the surgical insult (9, 10). Some studies have shown that elevated perioperative RPP is associated with poor prognosis (11–13). However, there is no research reporting the relationship between RPP and postoperative POD yet. The physiological tumult experienced by an elderly patient immediately following a hip fracture could understandably trigger a significant sympathetic surge, the magnitude of which, as captured by RPP, might offer a window into their subsequent resilience or vulnerability to cerebral dysfunction (14).

Concurrently, the role of systemic inflammation in the pathogenesis of POD is increasingly recognized as a critical axis (15, 16). Interleukin-1β (IL-1β) and Interleukin-6 (IL-6), are key mediators in this process (17–19). IL-1β is a potent pyrogen known to readily cross the blood–brain barrier, inducing neuroinflammatory changes that can precipitate delirium (20, 21). IL-6, a pleiotropic cytokine, stands as a sentinel marker of this acute-phase reaction and has been implicated in various neurological and psychiatric conditions, including delirium (22). Both cytokines can act synergistically to disrupt neuronal function (23). Given their central and well-documented roles, numerous studies have linked elevated preoperative levels of IL-1β and IL-6 to POD across different surgical populations (22, 24–26). Therefore, investigating these two specific cytokines provides a focused yet powerful lens through which to examine the inflammatory mediation pathway. However, the level of these cytokines in plasma under high RPP conditions has not been studied yet.

Therefore, this study aimed to explore the relationship between RPP at admission and incidence of POD in elderly patients undergoing hip fracture surgery. More innovatively we sought to unravel the intricate relationship between these variables by examining whether the impact of admission RPP on POD is mediated through inflammatory response.

## 2 Methods

### 2.1 Study design and participants

This retrospective cohort study was conducted at Beijing Jishuitan Hospital, a tertiary care center in Beijing, China. This research was ratified by the Ethics Committee of Peking University Third Hospital (IRB00006761-M2023560) and registered with the Chinese Clinical Trials database (ChiCTR2300077639). The inclusion criteria were elderly patients aged 65 and above with unilateral hip fractures and underwent surgical repair (internal fixation or arthroplasty) under spinal anesthesia between November 2021 and April 2022. Exclusion criteria were: (1) preexisting delirium or dementia; (2) multiple trauma or pathological fractures; (3) uncontrolled hypertension or arrhythmias; (4) severe sensory impairment precluding assessment; (5) inability to provide informed consent due to severe cognitive impairment. (6) preoperative or intraoperative using of anticholinergic drugs and polypharmacy.

### 2.2 Data collection and definitions

We collected the basic characteristics of the patients including age, sex, body mass index (BMI), education level, comorbidities, American Society of Anesthesiologists (ASA) physical status, and preoperative medication use. Cognitive function was assessed using the Mini-Mental State Examination (MMSE) at admission. Functional status was evaluated using the Barthel Index. The patient’s sleep status was evaluated using the Pittsburgh Sleep Quality Index (PSQI). And evaluated the patient’s overall condition using age adjusted Charlson compliance index (ACCI) scores. Vital signs, including heart rate and blood pressure, were recorded. Furthermore, a comprehensive panel of preoperative laboratory data was extracted from the electronic medical records, including complete blood count (WBC, RBC, PLT), liver function tests (ALT, AST), renal function (Creatinine), nutritional status (Albumin), bone metabolism markers (tP1NP, *β*-CTX, PTH), endocrine function (TSH, 25-OHVD), and arterial blood gas analysis (PaO2). These variables were included to provide a holistic view of the patients’ baseline physiological state.

### 2.3 Measurement of RPP

The RPP, a surrogate for myocardial workload and sympathetic activity, was calculated as the product of heart rate (beats per minute, bpm) and systolic blood pressure (mmHg). These vital signs were non-invasively measured using an automated oscillometric device under standardized conditions (after at least 5 min of rest, in a supine or sitting position). RPP was determined at three critical time points: upon hospital admission (within the first few hours), on the morning of surgery (preoperative morning), and on the first postoperative day (during routine morning vital sign assessment, typically between 8:00 and 9:00 a.m.).

### 2.4 Assessment of POD

POD was diagnosed using the 3-Minute Diagnostic Interview for CAM-defined Delirium (3D-CAM) assessment tool (27). It has high sensitivity and specificity and can be completed within 3 min (28). The patients were evaluated by a geriatric doctor trained in professional psychiatry at 8:00 and 20:00 every day for 2 days after surgery. Delirium severity measured by the Memorial Delirium Assessment Scale (MDAS).

### 2.5 Inflammatory biomarker assays (IL-1β, IL-6)

Venous blood samples were drawn into EDTAK2-containing tubes (BD Biosciences, San Jose, CA, USA) at admission. Plasma was promptly separated by centrifugation at 4,000 rpm for 10 min at 4 °C and stored frozen at −80 °C until batch analysis. Plasma concentrations of IL-1β and IL-6 were quantified using commercially available high-sensitivity enzyme-linked immunosorbent assay (ELISA) kits (Boster, Wuhan, China), strictly following the manufacturer’s protocols. All assays were performed in duplicate to ensure precision.

### 2.6 Anesthesia and analgesia management

All patients received standard perioperative care based on an established clinical pathway. Each patient underwent radial artery puncture catheterization to monitor invasive arterial pressure, non-invasive blood pressure, five lead electrocardiogram, and blood oxygen saturation. Before performing the spinal anesthesia position, each patient underwent iliac fascial space block on the affected side and was given 30 mL of 0.4% ropivacaine. Spinal anesthesia was performed with 0.5% bupivacaine (7.5–12.5 mg) injected into the L2-L3 or L3-L4 interspace. Each patient used a pain pump after surgery. Drug formula in pain pump was as follow: sufentanil 1 μg/kg, flurbiprofen axetil 200 mg, tropisetron hydrochloride 10 mg and normal saline to a volume of 100 mL. If the patient still feels significant pain, implement a remedial analgesia plan: oral acetaminophen and as-needed intravenous duromeprazole, targeting a NRS (numerical rating scale) pain score <4. The use of rescue analgesia was systematically recorded for all patients.

### 2.7 PSM

To achieve a more robust and statistically sound comparison between the POD and non-POD groups, a sophisticated PSM strategy was meticulously employed. Recognizing the profound and well-documented influence of advancing age, pre-existing cognitive status (as measured by MMSE), and overall comorbidity burden (quantified by ACCI) on the incidence of POD, we performed a 1:5 matching of patients who developed POD to those who did not (non-POD). This matching was predicated on these three key baseline variables: age, MMSE score, and ACCI score. The propensity scores, representing the estimated probability of being in the POD group given these baseline covariates, were calculated using a multivariable logistic regression model. A radius matching algorithm was utilized, with a defined caliper width set at 0.25 of the standard deviation of the logit of the propensity score. This careful and statistically rigorous process was specifically designed to achieve a balanced distribution of these critical potential confounders (age, MMSE, and ACCI) between the POD and non-POD groups, thereby substantially enhancing the internal validity and credibility of our subsequent comparative analyses. The specifics of the matching procedure, including success rates, are detailed in Supplementary Table S1. The effectiveness of the PSM in achieving balance for age, MMSE, and ACCI was subsequently confirmed through statistical testing, as presented in Supplementary Table S2. This process resulted in a final matched cohort of 25 POD patients and 125 non-POD patients.

### 2.8 Statistical analysis

The statistical analyses were executed using SPSS version 26.0 (IBM Corp., Armonk, NY, USA) and R software version 4.1.2 (R Foundation for Statistical Computing, Vienna, Austria). Continuous variables were presented as mean ± standard deviation (SD) for normally distributed data or median (interquartile range, IQR) for non-normally distributed data, as determined by the Shapiro–Wilk test. Categorical variables were expressed as counts and percentages (*n* %).

For comparisons between the POD and non-POD groups post-PSM, Student’s *t*-test or the Mann–Whitney U test was employed for continuous variables, as appropriate. The Chi-square test or Fisher’s exact test was utilized for categorical variables. A two-tailed *p*-value < 0.05 was considered statistically significant.

We developed a binary logistic regression model to assess the association between RPP at admission and the risk of POD. POD and RPP at admission were considered as dependent and independent variables, respectively. Odds ratios (ORs) with their corresponding 95% confidence intervals (CIs) were calculated.

The predictive performance of admission RPP for POD was evaluated using receiver operating characteristic (ROC) curve analysis. The area under the ROC curve (AUC) with its 95% CI was calculated, and an optimal cut-off value was determined by maximizing the Youden index (Sensitivity + Specificity − 1).

To delve into the mechanistic underpinnings, a mediation analysis was conducted. The PROCESS macro for SPSS (Model 4) developed by Hayes was utilized, employing bootstrapping with 5,000 resamples to estimate the direct effect (c’), indirect effect (a*b), and total effect (c). The significance of the indirect effect was determined by the absence of zero within the 95% bias-corrected bootstrap CI.

## 3 Results

### 3.1 Patient enrollment and characteristics post-PSM

**Figure 1:**
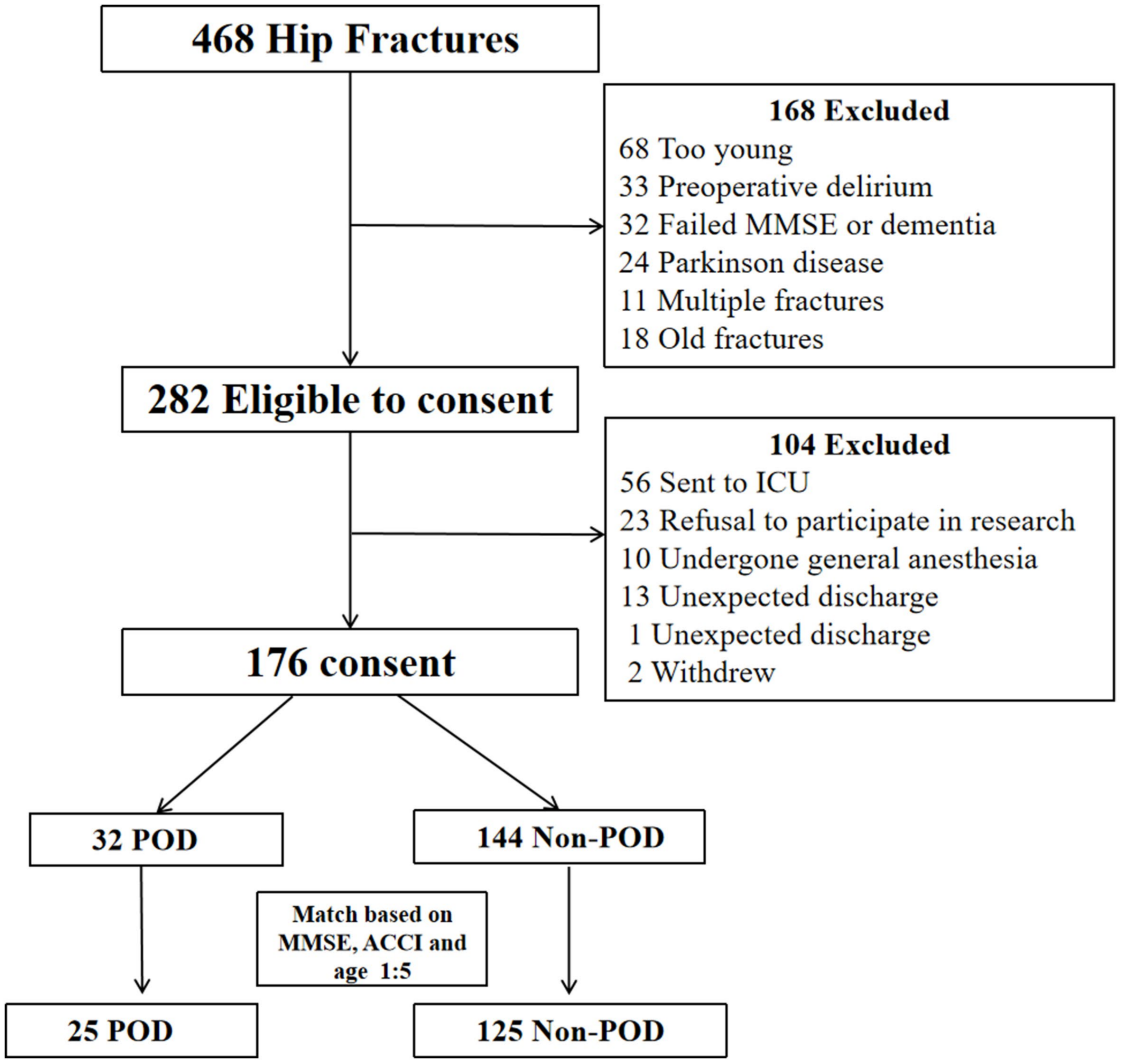
Flow diagram of patient screening, enrollment, and group allocation.

The journey of patient selection, from initial screening to the constitution of the final propensity score-matched cohort, is meticulously charted in Figure 1. Following the rigorous application of predefined inclusion and exclusion criteria, and the subsequent sophisticated PSM procedure, a final cohort comprising 150 elderly hip fracture patients formed the solid bedrock of our subsequent analyses. This cohort was composed of 25 individuals who were subsequently diagnosed with POD and 125 individuals who remained non-POD.

A pivotal achievement of the PSM process was the successful and statistically verified balancing of the matching variables: age (POD: 81.00 [75.0, 86.0] years vs. non-POD: 78.00 [71.3, 83.0] years, *p* = 0.112), baseline MMSE score (POD: 25.00 [24.0, 25.0] points vs. non-POD: 25.00 [24.0, 26.0] points, *p* = 0.072), and ACCI score (POD: 5.00 [4.0, 6.0] points vs. non-POD: 4.00 [4.0, 5.0] points, *p* = 0.259) (Table 1).

**Table 1:** Comparison of basic characteristics and preoperative laboratory tests between POD group and non-POD group patients before and after propensity score matching (PSM).

	Before PSM				After PSM			
	Non-POD group	POD group	t/χ^2^/z	*p*	Non-POD group	POD group	t/χ^2^/z	*p*
Age (years)	76.000 (70.0, 82.0)	82.000 (78.3, 87.5)	−3.636	<0.001**	78.000 (71.3, 83.0)	81.000 (75.0, 86.0)	−1.589	0.112
Sex (female)	109 (75.69)	22 (68.75)	0.663	0.415	92 (74.19)	17 (68.00)	0.406	0.524
BMI (kg/m^2^)	23.38 ± 3.78	23.15 ± 2.96	0.312	0.756	23.38 ± 3.85	23.43 ± 3.05	−0.065	0.948
ASA physical status class								
II	91 (63.19)	21 (65.63)	0.502	0.479	74 (59.68)	15 (60.00)	0.001	0.976
III	53 (36.81)	11 (34.38)			50 (40.32)	10 (40.00)		
Education (years)	9.000 (6.0, 12.0)	9.000 (5.0, 15.0)	−0.313	0.755	9.000 (6.0, 12.0)	9.000 (6.0, 15.0)	−0.872	0.383
MMSE score (points)	25.000 (24.3, 26.0)	24.000 (24.0, 25.0)	−3.673	<0.001**	25.000 (24.0, 26.0)	25.000 (24.0, 25.0)	−1.799	0.072
ADL score (points)	100.000 (90.0, 100.0)	97.500 (85.0, 100.0)	−1.027	0.304	100.000 (90.0, 100.0)	100.000 (85.0, 100.0)	−0.056	0.956
PSQI score (points)	13.24 ± 5.67	15.19 ± 5.21	−1.780	0.077	13.54 ± 5.50	15.20 ± 5.19	−1.389	0.167
ACCI score (points)	4.000 (3.0, 5.0)	5.000 (4.0, 6.0)	−2.221	0.026*	4.000 (4.0, 5.0)	5.000 (4.0, 6.0)	−1.129	0.259
RPP at admission (×10^3^ bpm·mmHg)	11.465 (9.9, 12.5)	12.920 (12.2, 13.9)	−4.010	<0.001**	11.357 (9.8, 12.5)	13.104 (12.5, 14.3)	−3.950	<0.001**
RPP at preoperative morning (×10^3^ bpm·mmHg)	11.522 (10.1, 12.5)	12.009 (11.0, 13.2)	−1.676	0.094	11.465 (10.1, 12.5)	11.679 (10.6, 12.9)	−0.922	0.356
RPP at postoperative day one (×10^3^ bpm·mmHg)	10.096 (8.9, 12.0)	11.157 (10.1, 12.1)	−1.801	0.072	10.197 (8.9, 12.0)	11.218 (9.9, 12.0)	−1.265	0.206
Injury-admission time (hours)	16.500 (6.0, 30.8)	13.000 (7.0, 38.5)	−0.188	0.851	13.000 (6.0, 30.0)	11.000 (7.0, 33.0)	−0.177	0.860
Smoke	9 (6.25)	4 (12.50)	1.495	0.221	9 (7.26)	3 (12.00)	0.632	0.427
Hypertension	68 (47.22)	21 (65.63)	3.547	0.060	58 (46.77)	15 (60.00)	1.456	0.228
Diabetes	41 (28.47)	8 (25.00)	0.157	0.692	38 (30.65)	8 (32.00)	0.018	0.894
Ischemic heart disease	35 (24.31)	8 (25.00)	0.007	0.934	33 (26.61)	8 (32.00)	0.303	0.582
Chronic obstructive pulmonary disease	8 (5.56)	1 (3.13)	0.319	0.572	7 (5.65)	1 (4.00)	0.111	0.739
Stroke	29 (20.14)	3 (9.38)	2.039	0.153	26 (20.97)	2 (8.00)	2.293	0.130
Using β-blockers	17 (11.81)	3 (9.38)	0.154	0.695	15 (12.10)	2 (8.00)	0.345	0.557
Using calcium channel blocker	37 (25.69)	11 (34.38)	0.995	0.319	32 (25.81)	8 (32.00)	0.406	0.524
Using ACEI/ARB	29 (20.14)	8 (25.00)	0.373	0.542	26 (20.97)	7 (28.00)	0.597	0.440
Using statins	31 (21.53)	8 (25.00)	0.183	0.669	28 (22.58)	8 (32.00)	1.007	0.316
Using insulin/hypoglycemic drugs	42 (29.17)	6 (18.75)	1.432	0.231	39 (31.45)	6 (24.00)	0.548	0.459
Preoperative laboratory examination								
Preoperative plasma IL-1β (pg/ml)	39.709 (27.5, 66.9)	41.299 (29.5, 64.6)	−0.056	0.956	41.168 (28.7, 66.5)	34.635 (26.6, 67.1)	−0.460	0.646
Preoperative plasma IL-6 (pg/ml)	7.750 (0.4, 16.9)	44.560 (21.7, 72.0)	−6.101	<0.001**	9.330 (0.5, 17.5)	42.670 (22.1, 69.1)	−5.507	<0.001**
WBC (×10^9^/L)	10.16 ± 3.17	11.14 ± 2.62	−1.600	0.112	9.81 ± 3.00	10.49 ± 1.55	−1.625	0.109
RBC (×10^12^/L)	4.09 ± 0.58	4.05 ± 0.61	0.328	0.743	4.03 ± 0.60	4.05 ± 0.47	−0.183	0.855
PLT (×10^9^/L)	195.500 (159.5, 231.8)	205.000 (159.0, 239.0)	−0.571	0.568	182.000 (156.5, 226.0)	195.000 (153.0, 227.5)	−0.290	0.772
ALT (U/L)	16.000 (13.0, 21.0)	15.000 (12.0, 33.0)	−0.271	0.786	15.000 (13.0, 20.5)	17.000 (13.5, 33.0)	−0.995	0.320
AST (U/L)	21.000 (17.0, 25.0)	21.000 (18.0, 27.0)	−0.720	0.471	21.000 (16.5, 25.0)	21.000 (18.0, 26.5)	−0.449	0.654
tP1NP (ug/L)	41.250 (33.5, 54.0)	48.450 (31.1, 62.8)	−1.074	0.283	40.900 (32.5, 54.2)	50.000 (33.7, 63.5)	−1.474	0.141
β-CTX (pg/ml)	0.380 (0.3, 0.6)	0.450 (0.3, 0.7)	−1.621	0.105	0.375 (0.3, 0.5)	0.440 (0.3, 0.6)	−1.243	0.214
PTH (pg/ml)	51.350 (37.1, 64.8)	57.250 (38.6, 78.8)	−1.167	0.243	52.650 (36.6, 66.3)	60.200 (39.0, 81.5)	−1.095	0.273
Albumin (g/L)	41.000 (38.7, 43.0)	41.000 (38.8, 42.9)	−0.173	0.863	40.900 (38.4, 42.6)	41.100 (39.0, 42.9)	−0.485	0.627
PaO2 (mmHg)	80.000 (71.3, 90.5)	78.000 (73.4, 84.0)	−0.929	0.353	79.000 (71.0, 88.0)	78.000 (71.5, 84.0)	−0.674	0.500
Blood glucose (mmol/L)	7.900 (6.7, 9.7)	7.600 (6.7, 9.1)	−0.804	0.422	8.000 (6.9, 9.9)	7.700 (6.8, 9.3)	−0.799	0.424
TSH (mIU/L)	1.675 (0.9, 2.7)	1.395 (0.9, 2.6)	−0.556	0.578	1.690 (0.9, 2.8)	1.345 (1.0, 2.5)	−0.696	0.486
25-OHVD (ng/ml)	14.700 (9.7, 19.6)	14.845 (10.7, 20.1)	−0.261	0.794	14.360 (9.8, 19.9)	16.050 (9.1, 20.5)	−0.341	0.733
Creatinine (mmol/L)	60.000 (51.0, 75.8)	65.000 (48.0, 86.0)	−0.812	0.417	61.000 (51.0, 78.5)	65.000 (48.0, 86.0)	−0.307	0.759
Use of rescue analgesia, *n* (%)	23 (16.0)	7 (21.9)	0.817	0.366	18 (14.4)	5 (20.0)	0.531	0.466

BMI, Body Mass Index; ASA, American Society of Anesthesiologists Classification; MMSE, Simplified Intelligence State Scale; ADL, Daily Living Ability Score; PSQI, Pittsburgh Sleep Quality Index; ACCI, Age Corrected Chalson’s Comorbidities Index; RPP, rate pressure product; WBC, white blood cells; RBC, red blood cells; PLT, platelets; ALT, alanine transaminase; AST, cereal grass transaminase; TSH, thyroid-stimulating hormone; PaO_2_, oxygen partial pressure.

However, after this rigorous matching, two crucial distinctions persisted with statistical significance. Patients in POD group presented with a significantly higher RPP at admission and elevated levels of preoperative plasma IL-6 (Table 1). It is also noteworthy that, as detailed in Table 1, other preoperative laboratory parameters, including complete blood count, liver and renal function, and nutritional markers, showed no significant differences between the POD and non-POD groups post-PSM. This finding suggests that the increased risk of POD is specifically associated with the initial stress response (RPP) and inflammatory activation (IL-6), rather than general physiological derangements.

### 3.2 Association of RPP with POD

As meticulously detailed in Table 1 (post-PSM data), the RPP at admission was found to be strikingly and significantly higher in those elderly patients who subsequently succumbed to the neuropsychiatric complication of POD, when compared to their counterparts who remained free of delirium (POD group: 13.104 [12.5, 14.3] × 10^3^ bpm·mmHg vs. non-POD group: 11.357 [9.8, 12.5] × 10^3^ bpm·mmHg, *p* < 0.001).

**Figure 2:**
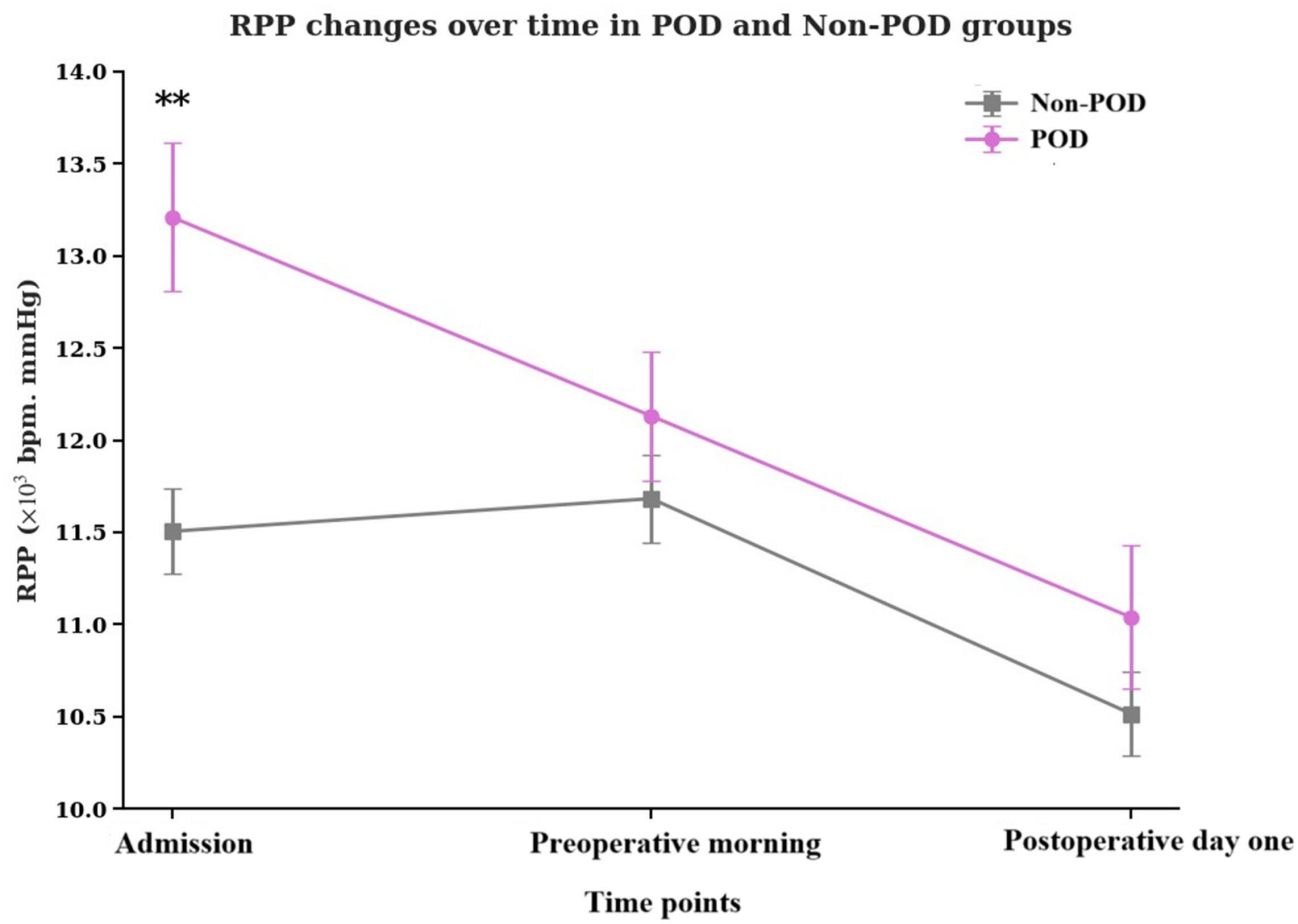
Temporal changes in the product of RPP at admission, preoperative morning, and postoperative day one in POD group patients and non-POD group patients. RPP, rate pressure product; POD, postoperative delirium.

The temporal dynamics of RPP across the perioperative period are vividly illustrated in Figure 2. In both groups, RPP tended to decrease from admission through the postoperative period, yet the POD group consistently maintained a higher trajectory, especially at the initial assessment. The RPP at admission of POD group patients was significantly higher than that of non-POD group patients (*p* < 0.001).

### 3.3 Perioperative inflammatory cytokine profiles in POD

**Figure 3:**
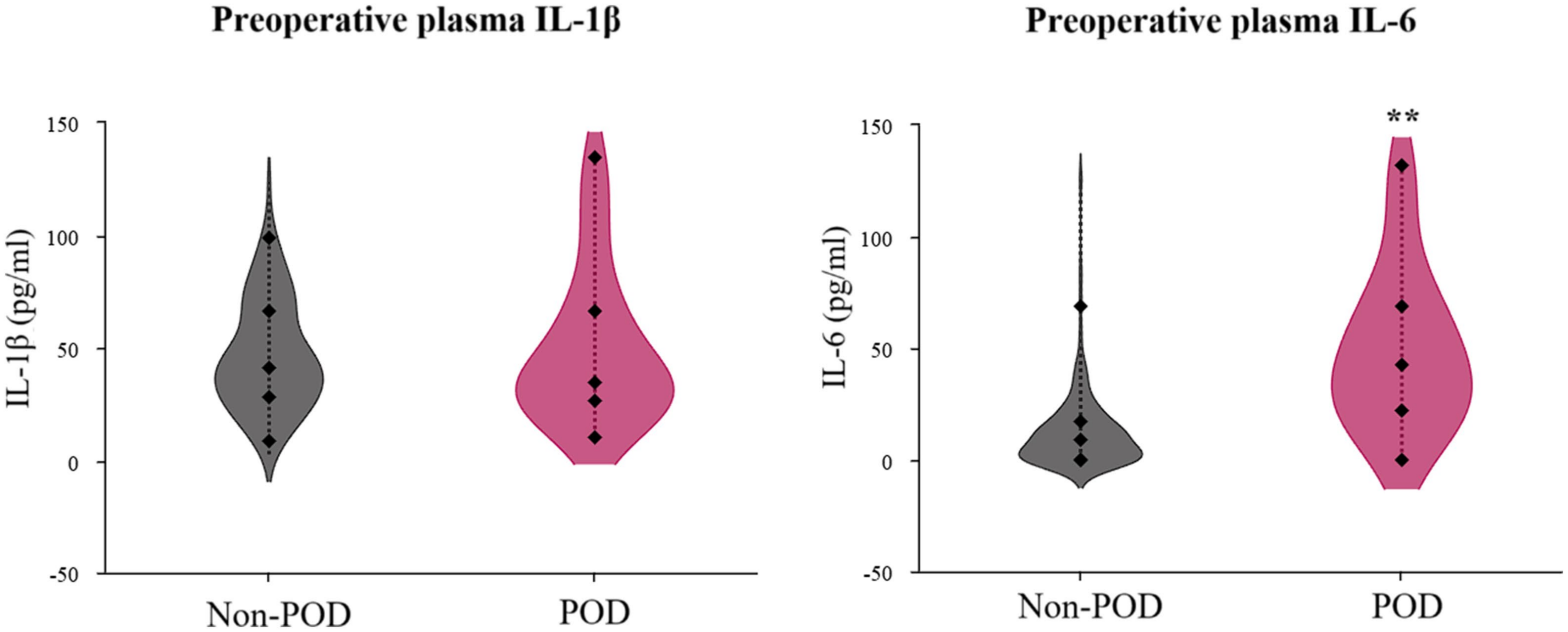
Violin plots illustrating preoperative plasma IL-1β and IL-6 concentrations in POD group patients and non-POD group patients. IL-1, interleukin-1; IL-6, interleukin-6; POD, postoperative delirium.

The examination of preoperative inflammatory markers unveiled a stark contrast in IL-6 levels, as depicted in Figure 3 and quantified in Table 1 (post-PSM data). Patients in the POD group exhibited dramatically and significantly elevated preoperative plasma IL-6 concentrations compared to the non-POD group (*p* < 0.001). In intriguing contrast, preoperative plasma IL-1β levels did not significantly differ between the two groups (*p* = 0.612).

### 3.4 Multivariable logistic regression analysis for POD

**Figure 4:**
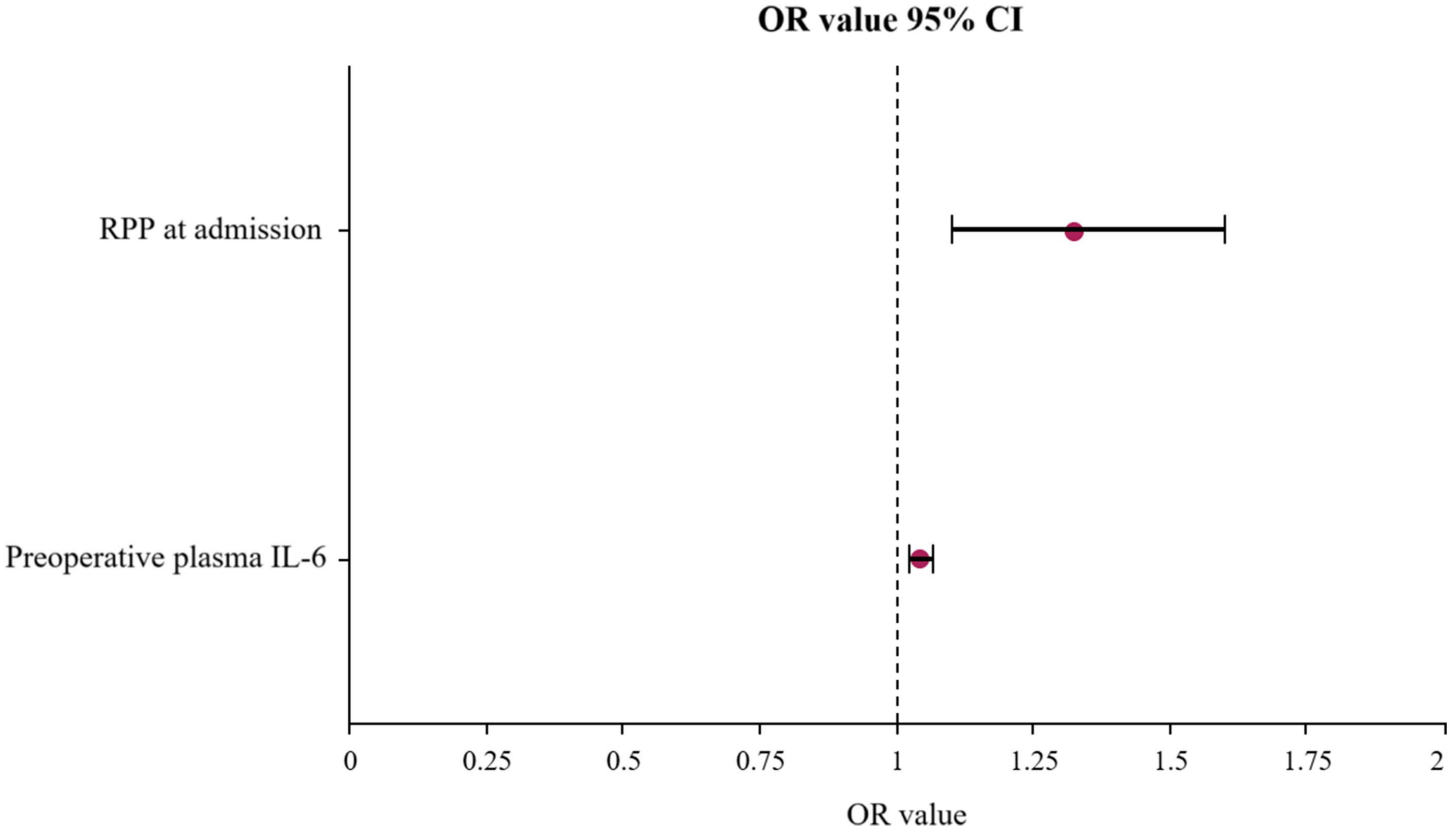
Forest plot displaying the OR and 95% CI for independent risk factors of POD identified by multivariate logistic regression analysis. OR, odds ratios; CI, confidence intervals; POD, postoperative delirium.

To dissect the independent contributions of various factors to POD development, a multivariate logistic regression analysis was performed. The results, presented in Table 2 and visualized in the forest plot in Figure 4. Elevated RPP at admission (expressed in ×10^3^ bpm·mmHg) was found to significantly and independently increase the likelihood of a patient developing POD. For each unit increase in admission RPP (×10^3^ bpm·mmHg), the odds of developing POD increased by approximately 32.5% (OR: 1.325, 95% CI: 1.098–1.599, *p* = 0.003).

**Table 2:** Logistic regression analysis results.

	Regression coefficient	Standard error	*z* value	Wald *χ*^2^	*p* value	OR value	95% CI
RPP at admission	0.282	0.096	2.937	8.624	0.003	1.325	1.098 ~ 1.599
Preoperative plasma IL-6	0.042	0.010	4.165	17.347	<0.001	1.043	1.023 ~ 1.064
Intercept	−6.189	1.342	−4.613	21.275	<0.001	0.002	0.000 ~ 0.028

OR, odds ratio; 95% CI, 95% confidence interval; IL-6, interleukin-6; RPP, rate pressure product.

Increased preoperative plasma IL-6 were also powerfully and independently associated with a substantially greater risk of developing POD. For each unit (pg/ml) increase in IL-6, the odds of POD escalated by approximately 4.3% (OR: 1.043, 95% CI: 1.023–1.064, *p* < 0.001).

### 3.5 ROC curve for RPP at admission in predicting POD

**Figure 5:**
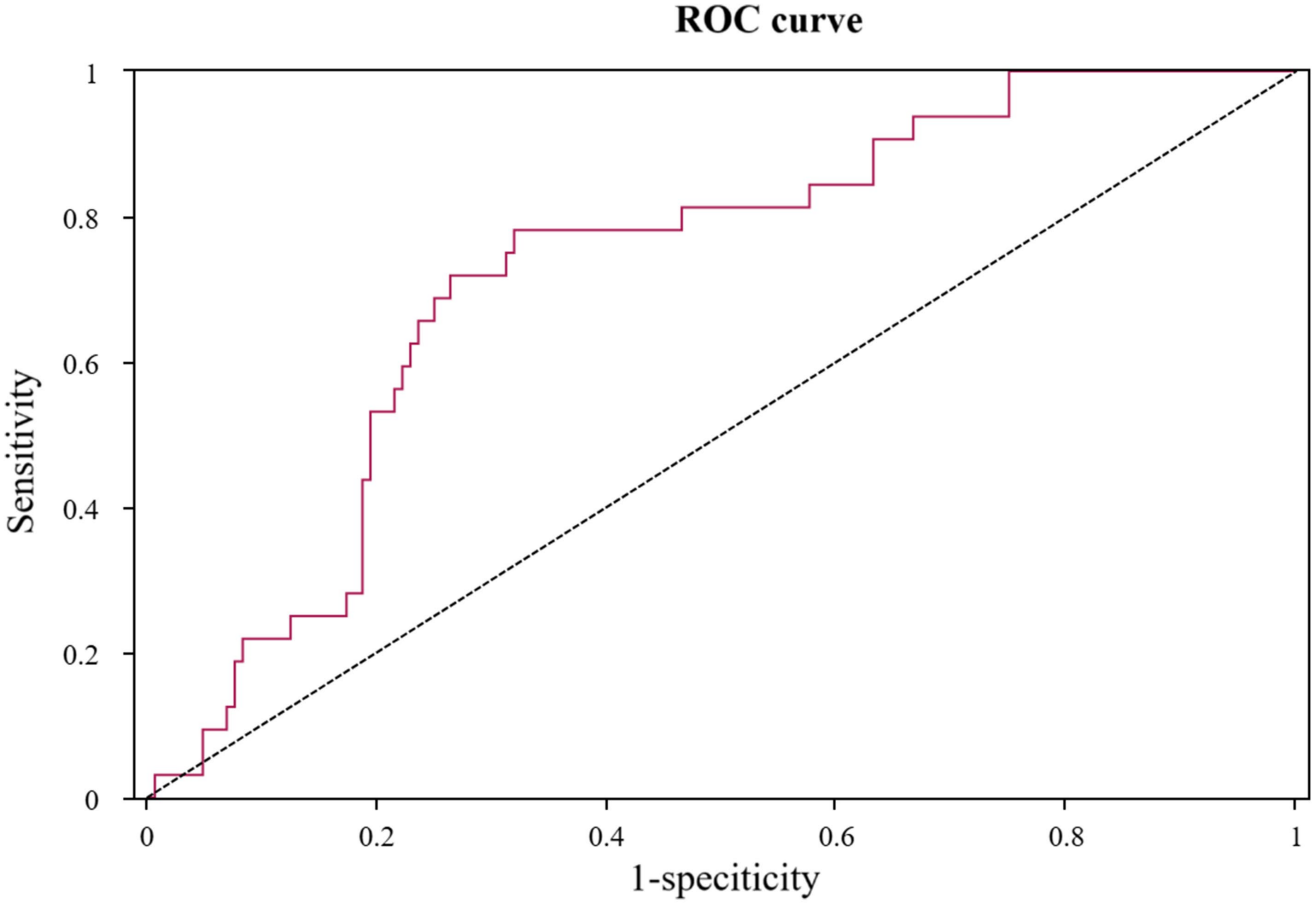
ROC curve for product of RPP at admission in predicting POD. ROC, receiver operating characteristic; RPP, rate pressure product; POD, postoperative delirium.

The utility of admission RPP as a prognostic tool for POD was further scrutinized using ROC curve analysis, detailed in Table 3 and illustrated in Figure 5. The analysis revealed that RPP at admission demonstrated a fair predictive accuracy for POD, with an AUC of 0.727 (95% CI: 0.639–0.815, *p* < 0.001). The optimal cut-off value for admission RPP, identified by maximizing the Youden index, was determined to be 12.136 × 10^3^ bpm·mmHg. At this threshold, the sensitivity was 0.781, and the specificity was 0.681. This suggests that a readily obtainable physiological parameter at the point of hospital entry holds tangible promise for risk stratification.

**Table 3:** The predicted values of PRR at admission for POD.

	AUC	Sensitivity + Specificity − 1	Sensitivity	Specificity	Cut-off	Std. error	*p*	95% CI for AUC
RPP at admission	0.727	0.462	0.781	0.681	12.136	0.045	<0.001**	0.639 ~ 0.815

AUC, area under the curve; 95% CI, 95% confidence interval; RPP, rate pressure product.

### 3.6 Mediation analysis of the relationship between RPP at admission, IL-6 and POD

The results of the mediation analysis, meticulously detailed in Table 4. The analysis revealed a significant total effect (path c) of RPP at admission on POD (Effect = 0.036, *p* = 0.002). Crucially, there was a significant indirect effect (path ab) of RPP at admission/100 on POD through preoperative plasma IL-6 (Indirect Effect = 0.014, 95% Bootstrap CI: 0.0032 to 0.032), as the confidence interval did not include zero. This indicates that the influence of elevated admission RPP on the likelihood of developing POD is, at least in part, channeled through an increase in preoperative IL-6 levels. The direct effect (path c’) of RPP at admission on POD, after accounting for the mediating role of IL-6, remained significant (Effect = 0.022, *p* = 0.031).

**Table 4:** Intermediary role test of the relationship between RPP at admission.

	Symbol	Symbol	Effect	95% CI		Standard error	*z-*value/*t-*value	*p-*value	Conclusion
				Lower limit	Upper limit				
RPP at admission ≥ Preoperative plasma IL-6 ≥ POD	a*b	Indirect effect	0.014	0.032	0.185	0.039	0.358	0.720	Partial mediation
RPP at admission ≥ Preoperative plasma IL-6	a	X ≥ M	1.917	0.306	3.528	0.822	2.332	0.021	
Preoperative plasma IL-6 ≥ POD	b	M ≥ Y	0.007	0.005	0.009	0.001	7.396	<0.001	
RPP at admission ≥ POD	c’	Direct effect	0.022	0.002	0.042	0.010	2.179	0.031	
RPP at admission ≥ POD	c	Total effect	0.036	0.013	0.059	0.012	3.118	0.002	

Perioperative plasma IL-6 and POD. RPP, rate pressure product; POD, postoperative delirium; IL-6, interleukin-6.

## 4 Discussion

The result of this study indicates that elevated RPP at admission is a risk factor of POD in elderly patients undergoing hip fracture surgery and the effect of RPP at admission on POD may be partially mediated by preoperative plasma IL-6.

A cardinal observation from our study is the robust and independent association between an elevated RPP upon hospital admission and an increased risk of subsequent POD. This simple, non-invasive metric, reflecting the heart’s workload and indirectly mirroring sympathetic nervous system activity (29), emerged as a significant harbinger of delirium. This finding mechanistically points to the pivotal role of the acute stress response. A hip fracture represents a profound physiological and psychological trauma, triggering an immediate surge in the sympathetic-adrenal-medullary (SAM) axis activity. This results in the release of catecholamines, which directly elevate heart rate and systolic blood pressure, manifesting as a high RPP (30–32). Crucially, this sympathetic storm does not merely exert peripheral effects; it is a systemic event with profound central nervous system implications. The resulting catecholamines can disrupt a vulnerable blood–brain barrier, leading to direct neurotoxic effects and altered cerebral perfusion, thereby creating a fertile ground for delirium to develop (33–35). There is currently no research on the relationship between RPP and POD. However studies have shown a correlation between preoperative heart rate variability (HRV), a parameter of the autonomic nervous system activity (ANSA) and the occurrence of POD (36–38). The acute stress of a hip fracture, often compounded by pain, anxiety, and immobility, can undoubtedly trigger a substantial sympathetic surge (39–41). The observation that RPP at admission, rather than later perioperative measurements, held the strongest association underscores the critical importance of the patient’s physiological state at the very point of entry into the acute care pathway.

Our study result suggests that higher plasma IL-6 levels upon preoperative admission are a risk factor for POD. Patients who developed delirium exhibited profoundly elevated preoperative plasma IL-6 levels compared to their non-delirious counterparts. This potent pro-inflammatory cytokine has been consistently implicated in the neuroinflammatory processes thought to underpin delirium, potentially by disrupting blood–brain barrier integrity, altering neurotransmitter systems, and directly impacting neuronal function (22, 42, 43). Previous studies have shown that higher levels of IL-6 in preoperative plasma are associated with postoperative delirium, which is consistent with our research findings (22, 26, 44).

Our study result indicates that the effect of RPP on POD at admission may be partially mediated by IL-6. This suggests a deleterious chain of events: the initial heightened sympathetic drive, manifested as an elevated RPP, does not merely act in isolation but also appears to fuel or exacerbate the subsequent IL-6 inflammatory cascade, which in turn significantly contributes to the development of delirium. The biological underpinning for this neuro-immune crosstalk is well-documented. Sympathetic nerve activation, through the release of norepinephrine, can directly modulate immune cell function via *β*-adrenergic receptors (45–47). In the acute phase of stress, this interaction often skews the immune response toward a pro-inflammatory state, promoting the synthesis and release of IL-6 from macrophages and other immune cells (48–52). This establishes a vicious cycle where heightened sympathetic tone and inflammation reinforce each other, magnifying the initial insult and leading to sustained neuroinflammation (53). Studies have shown that IL-6 levels increase under sympathetic nervous system activation (54, 55).

Furthermore, the results of our mediation analysis provide a nuanced perspective. The significant indirect effect through IL-6 validates this specific inflammatory pathway. However, the persistence of a significant direct effect of RPP on POD is equally illuminating. It strongly implies that the detrimental impact of sympathetic overactivation is not exclusively channeled through IL-6. Other mechanisms, such as direct catecholaminergic neurotoxicity (56), RPP-induced changes in cerebral autoregulation (57), or the involvement of other unmeasured inflammatory mediators (e.g., TNF-*α*, high-mobility group box 1) (58, 59), are likely at play. This partial mediation underscores the multifaceted pathophysiology of POD and highlights RPP as a composite indicator of multiple converging risk pathways.

The clinical implications of these findings are manifold and potentially far-reaching. The RPP at admission, being an easily calculable parameter from routinely measured vital signs, holds promise as an early, accessible, and cost-effective screening tool to identify patients at heightened risk for POD (60, 61). An admission RPP exceeding the identified cut-off of 12.136 × 10^3^ bpm·mmHg (AUC 0.727) could trigger a heightened state of vigilance and the proactive implementation of multicomponent delirium prevention strategies. Furthermore, the strong association with IL-6, and its mediating role, might open avenues for more targeted interventions. While broad anti-inflammatory strategies have yielded mixed results for POD, understanding the specific upstream drivers (like sympathetic overactivity) and downstream effectors (like IL-6) could refine therapeutic targets. Future research might explore whether interventions aimed at blunting the initial sympathetic surge in high-RPP patients could attenuate the subsequent IL-6 response and, ultimately, reduce POD incidence.

Our study possesses several notable strengths. The use of propensity score matching, specifically targeting three key confounders (age, baseline MMSE, and ACCI) using a 1:5 ratio, lending greater internal validity to the observed associations. The comprehensive assessment battery, including validated cognitive and functional scales, and the meticulous diagnosis of POD add to the robustness of our data. Furthermore, the sophisticated employment of formal mediation analysis to explore the intricate mechanistic interplay between RPP, IL-6, and POD represents a novel and insightful contribution to the evolving body of literature in this critical field of geriatric perioperative care.

Nevertheless, we must also acknowledge certain limitations of this study. First, the study was conducted at a single academic center with a relatively small sample size, which may limit the generalizability of our findings to other healthcare settings or patient populations with different baseline characteristics and may limit the generalizability of our findings. Second, unmeasured confounders, despite the rigor of PSM, may still lurk within the data, subtly influencing the observed relationships. Third, due to the retrospective design, this was recorded during routine morning rounds on the first postoperative day rather than at a standardized interval from the end of surgery. This variability in the time from surgical insult to measurement could introduce noise into the postoperative RPP data, although our primary finding relies on the robust admission RPP value. Future prospective studies should implement a standardized timing protocol to overcome this limitation. Fourth, while IL-6 is a key inflammatory marker, the inflammatory cascade is complex, and other cytokines or inflammatory pathways not measured in this study could also play significant roles. Last, we did not evaluate the patient’s fragile status and presence of sensory limitation of any degree. In future research, we will combine multidimensional assessment to establish a more comprehensive predictive model.

Future research endeavors should aim to validate these findings in larger, multicenter cohorts. Investigating the impact of interventions specifically targeting the reduction of admission RPP (e.g., through early pain management, anxiolysis) on IL-6 levels and POD incidence would be next step. Further exploration of the temporal dynamics of a broader panel of inflammatory and neuro-injury biomarkers in relation to RPP changes could also provide deeper insights into the physiological systems that culminates in delirium.

## 5 Conclusion

Elevated RPP at admission is a risk factor of POD in elderly patients undergoing hip fracture surgery and the effect of RPP at admission on POD may be partially mediated by preoperative plasma IL-6.

## Data Availability

The datasets presented in this study can be found in online repositories. The names of the repository/repositories and accession number(s) can be found in the article/Supplementary material.
