# Supplementary material for: Association between perioperative rate pressure product and postoperative delirium in geriatric patients with hip fracture
Source: Front Med (Lausanne). 2025 Oct 28;12:1651278. doi: 10.3389/fmed.2025.1651278 (PMC12602479; doi:10.3389/fmed.2025.1651278)
Supplement: Supplementary file 1 [file Table_1.docx]

| PSM basic information | |
| --- | --- |
|  | information |
| Matching mode | 1:5 matching |
| Matching method | Radius matching (precise matching priority) |
| Threshold (radius value or caliper value) | 0.25 |
| Sample method | sampling without replacement |
| Need to match a certain number | 32 |
| The average number of successful matches | 5.0 |
| Number of successful matches | 25 |
| Number of failed matches | 7 |
| Matching success rate | 78.125% |
| Match failure rate | 21.875% |

**Supplementary table 1.** Basic information for propensity score matching. Matching POD patients and non POD patients 1:5 based on age, MMSE, ACCI.
