# Supplementary material for: Association between perioperative rate pressure product and postoperative delirium in geriatric patients with hip fracture
Source: Front Med (Lausanne). 2025 Oct 28;12:1651278. doi: 10.3389/fmed.2025.1651278 (PMC12602479; doi:10.3389/fmed.2025.1651278)
Supplement: Supplementary file 2 [file Table_2.docx]

| PSM parallel hypothesis testing | | | | | | | |
| --- | --- | --- | --- | --- | --- | --- | --- |
|  |  | Treated (experimental group) | Control (control group) | Standardization deviation (%) | Reduction amplitude of standardization deviation (%) | *t* value | *p* value |
| Age | Before matching | 82.531 | 76.688 | 76.39% | 53.10% | 3.978 | 0.000 |
|  | After matching | 80.440 | 77.871 | 35.83% |  | 1.721 | 0.093 |
| MMSE | Before matching | 4.906 | 4.326 | 41.13% | 43.70% | 2.137 | 0.038 |
|  | After matching | 4.880 | 4.548 | 23.16% |  | 1.033 | 0.309 |
| ACCI | Before matching | 24.688 | 25.514 | -73.01% | 51.68% | -4.032 | 0.000 |
|  | After matching | 24.880 | 25.266 | -35.28% |  | -1.650 | 0.108 |

**Supplementary table 2.** Propensity score matching parallel hypothesis testing.
