# Supplementary material for: Association between perioperative rate pressure product and postoperative delirium in geriatric patients with hip fracture
Source: Front Med (Lausanne). 2025 Oct 28;12:1651278. doi: 10.3389/fmed.2025.1651278 (PMC12602479; doi:10.3389/fmed.2025.1651278)
Supplement: Supplementary file 3 [file Table_3.docx]

|  | | | | | | | | | | | | | | | |
| --- | --- | --- | --- | --- | --- | --- | --- | --- | --- | --- | --- | --- | --- | --- | --- |
| Variables | POD | | | | | Preoperative plasma IL-6 | | | | | POD | | | | |
|  | *B* | Standard error | *t* | *p* | *β* | *B* | Standard error | *t* | *p* | *β* | *B* | Standard error | *t* | *p* | *β* |
| Constant | -0.262 | 0.139 | -1.882 | 0.062 | - | -4.005 | 9.913 | -0.404 | 0.687 | - | -0.233 | 0.119 | -1.953 | 0.053 | - |
| RPP at admission | 0.036** | 0.012 | 3.118 | 0.002 | 0.250 | 1.917* | 0.822 | 2.332 | 0.021 | 0.190 | 0.022* | 0.010 | 2.179 | 0.031 | 0.152 |
| Preoperative plasma IL-6 |  |  |  |  |  |  |  |  |  |  | 0.007** | 0.001 | 7.396 | 0.000 | 0.516 |
| *R* 2 | 0.062 | | | | | 0.036 | | | | | 0.319 | | | | |
| Adjust *R* 2 | 0.056 | | | | | 0.029 | | | | | 0.310 | | | | |
| *F* value | *F* (1,146)=9.722,*p*=0.002 | | | | | *F* (1,146)=5.439,*p*=0.021 | | | | | *F* (2,145)=34.002,*p*=0.000 | | | | |
| * *p*<0.05 ** *p*<0.01 | | | | | | | | | | | | | | | |

**Supplementary table 3**. The mediation analysis of the relationship between RPP at admission, Perioperative plasma IL-6 and POD. B, non standardized regression coefficient; β, standardized regression coefficient; t, B/Standard error. RPP, Product of heart rate and systolic blood pressure. POD, postoperative delirium; IL-6, interleukin-6. * *p*<0.05 ** *p*<0.01. Bold values represent the *p*-value.
